# Cerebral Phaeohyphomycosis Caused by *Exophiala dermatitidis* in a Chinese *CARD9*-Deficient Patient: A Case Report and Literature Review

**DOI:** 10.3389/fneur.2019.00938

**Published:** 2019-09-03

**Authors:** Chen Wang, Hongyi Xing, Xiaobing Jiang, Jingsi Zeng, Zhijun Liu, Jixiang Chen, Yan Wu

**Affiliations:** ^1^Department of Neurology, Tongji Medical College, Union Hospital, Huazhong University of Science and Technology, Wuhan, China; ^2^Department of Neurosurgery, Tongji Medical College, Union Hospital, Huazhong University of Science and Technology, Wuhan, China; ^3^Department of Dermatology and Venereology, Tongji Medical College, Union Hospital, Huazhong University of Science and Technology, Wuhan, China

**Keywords:** *CARD9*, cerebral phaeohyphomycosis, *Exophiala dermatitidis*, fungal infection, loss-of-function mutation

## Abstract

*Exophiala dermatitidis*, a dematiaceous fungus typically found in decaying organic matter worldwide, is a rare cause of fungal infections. Cerebral phaeohyphomycosis is a sporadic but often fatal infection of the brain caused by *E. dermatitidis*. However, due to limited reports, little is known about its specific predisposing factors, clinical manifestation, and optimal treatment modality. Here, we report a clinical presentation and management of cerebral phaeohyphomycosis in a Chinese patient. An otherwise healthy, young male who was diagnosed with neck fungal lymphadenitis caused by *E. dermatitidis* 7 months prior and was treated with itraconazole, presented later with progressive intracranial hypertension and persistent coma. Culture of the neck lymphoid tissue produced growth of a black yeast-like fungus, which was identified as *E. dermatitidis* by sequencing of the ribosomal DNA internal transcribed spacer (ITS) domains. Accordingly, a cerebral biopsy was performed, and the pathological report showed mycelia and fungal granulomas. We also sequenced *CARD9* in the patient and found him to be homozygous for loss-of-function mutation; his parents were heterozygous for the same mutation. This is a first case report of cerebral phaeohyphomycosis caused by *E. dermatitidis* in a *CARD9*-deficient Chinese patient. He eventually succumbed to brain herniation and severe lung infection with a poor response to therapy. Thus, previously healthy patients with unexplained invasive *E. dermatitidis* infection, at any age, should be tested for inherited *CARD9* deficiency.

## Introduction

Over the past two decades, fungal infections of the central nervous system (CNS) have become more common. The invasion of the CNS depends largely on the host's immune state and the virulence of the fungal strain. The involvement of the CNS usually leads to fatal consequences. Cerebral phaeohyphomycosis is often a fatal disease caused by truly neurotropic dematiaceous fungi, characterized by black necrotic tissue, black pus, and black cerebrospinal fluid (CSF) (1). *Exophiala dermatitidis* (encountered worldwide, however, common in East Asia), also called *Wangiella dermatitidis*, is an emerging dematiaceous fungus associated with high mortality rates in both immunocompromised and competent hosts (2, 3). Cerebral phaeohyphomycosis is limited mostly to individuals of East Asian ethnicity. *CARD9* is a caspase recruitment domain (CARD)-containing protein recently reported to be involved in invasive fungal diseases (4). We present the first reported case of cerebral phaeohyphomycosis caused by *E. dermatitidis* in a *CARD9*-deficient Chinese patient.

## Case Presentation

**Figure 1:**
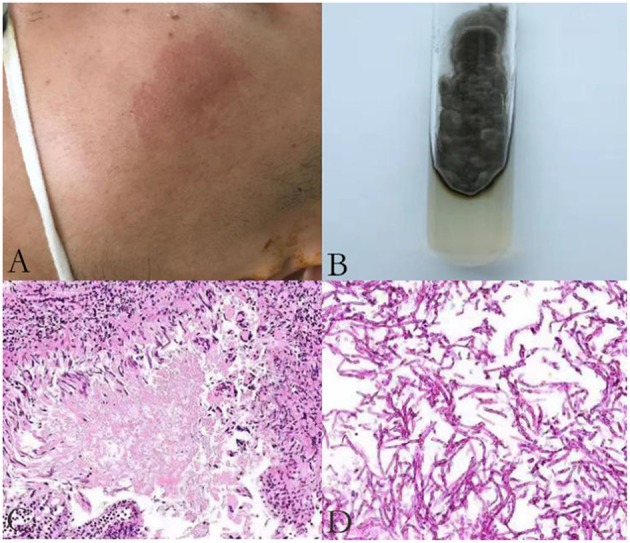
An itchy rash on the left side of patient's face **(A)**; A brown mold cultured in Sabouraud Dextrose Agar **(B)**; Histological features of the lymph node biopsy **(C–D)**; Hematoxylin-eosin H-E staining shows fungi surrounded by an epithelioid and giant cell granuloma (original magnification ×200) **(C)**; H-E staining demonstrates infiltration of fungi without granuloma to another area of the biopsy sample (original magnification ×400) **(D)**.

A 23 year old male was first admitted to our hospital with a history of an itchy rash present for more than 7 months on the left side of his face and multiple neck masses for 1 month, without fever, cough, sputum, headache, dizziness, diarrhea, and any other discomfort. He was in prison for more than 3 years and had been released recently. He had a history of a scissors puncture on his face before the onset of the facial lesion. The parents are consanguineous. The dark red patches observed on his face (Figure 1A) were about the size of an egg, soft, and well defined, with no ulceration. Many enlarged, hard, and mobile lymph nodes ranging from the size of soybeans to quail eggs with no tenderness were noticed on the skin behind the left ear and neck. The complete blood cell count, acute-phase reactants (erythrocyte sedimentation rate, serum C-reactive protein, and procalcitonin), serum biochemistry tests including glucose, and liver function tests were normal. The serologic tests for viruses (HIV, syphilis, and hepatitis virus) were negative. The serum antibodies (anti-double-stranded DNA antibodies, anti-neutrophil cytoplasmic antibodies, anti-ribonucleoprotein antibodies, and anti-Smith antibodies) were within the normal range. The cultures for blood bacteria and fungi were negative. The lymphocyte subsets showed no obvious abnormality. The titers of anti-nuclear antibodies were 1:100 (<1:100), anti-JO-1 antibodies were weakly positive, and blood IgE was 1693.10 IU/mL (normal 1–190 IU/mL). He did not present any symptoms of tuberculosis; however, the T-spot and purified protein derivative (PPD) skin tests were positive. He had a previous history of tuberculosis infection. Computed tomography (CT) of his lungs revealed a number of speckled shadows and nodules in both the lungs. The cranial CT showed no abnormality. Subsequently, cervical lymph node biopsy and culture of the neck lymphoid tissue was performed. The culture of the neck lymphoid tissue yielded a black yeast-like fungus (Figure 1B); the pathological report indicated fungal granulomatous inflammation with necrosis (Figures 1C,D), and the strain was later identified as *E. dermatitidis* by DNA sequencing of the ribosomal DNA ITS domains. A pair of primer was designed for the amplification and sequencing of the ribosomal DNA (ITS4: 5′-TCCTCCGCTTATTGATATGC-3′ ITS1: 5′-TCCGTAGGTGAACCTGCGG-3′). The ITS sequence was found to be identical to that of *Exophiala dermatitidis* type strain CBS 207.35 (GenBank nr_121268.1). The patient was discharged and prescribed an anti-fungal medication, itraconazole, and his facial rash improved over time.

**Figure 2:**
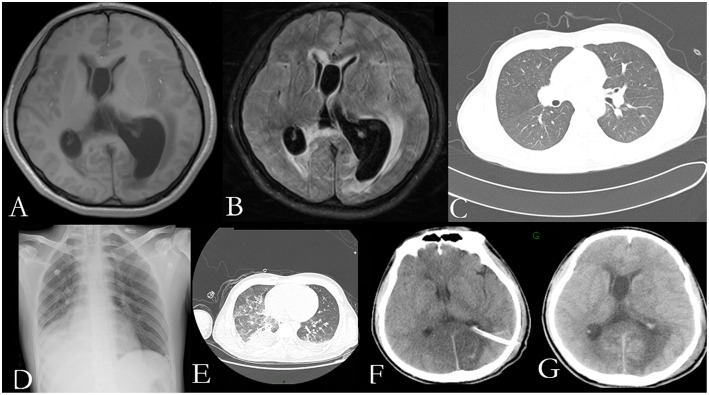
Radiological examination of the patient: T1 weighted axial and T2-flair brain MR imaging showing pachymeningitis and hydrocephalus **(A,B)**; The lung CT on the first day of his second admission shows no obvious signs of inflection **(C)**; The chest radiograph **(D)**; and lung CT **(E)** show pulmonary infection, pleural effusion, and partial atelectasis; Head CT on the first day after surgery **(F)** and a week after surgery **(G)** show rapid accumulation of cerebrospinal fluid (CSF).

**Figure 3:**
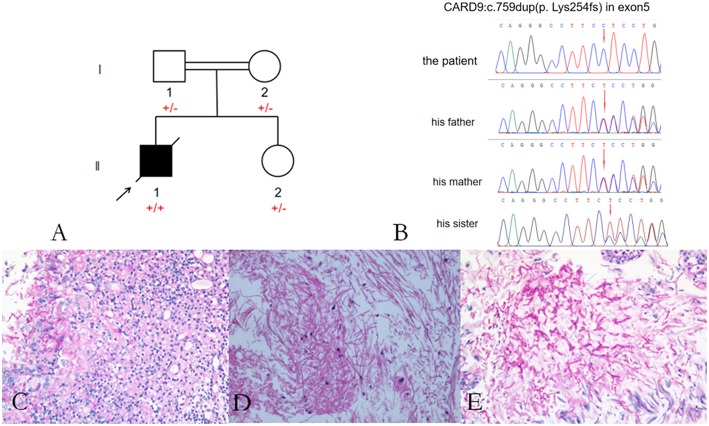
Pedigree of the patient with *Exophiala dermatitidis* infection and a *CARD9* mutation: Square indicates male; circle indicates female; filled symbol indicates affected individual; diagonal line across the symbol indicates deceased individual; arrow indicates the proband **(A)**; A novel c.759dup (p. Lys254fs) mutation was identified in the exon 5 of *CARD9* **(B)**; Histological features of the brain tissue biopsy **(C–E)**: Hematoxylin-eosin H-E staining shows fungal granuloma **(C)**, magnification ×200] and mycelia [**(D)**, magnification ×400]. PSA staining shows mycelia **(E)**. Abbreviations are as follows: +, mutation; –, wild-type.

He was again admitted to our hospital 7 months later with headache, nausea, and vomiting persistent for a week, accompanied with no convulsions, unconsciousness, fever, and paralysis. A brain CT done in a local hospital suggested hydrocephalus and cerebral hernia. His vital signs at the time of admission were stable. Physical examination disclosed no obvious abnormality except neck stiffness. There was a slight decrease in the white and red blood cell counts and an insignificant increase in the percentage of monocytes. The serum biochemistry test revealed no significant abnormality. Compared with the previous results, the blood IgE was reduced to 938.20 IU/mL (normal 1–190 IU/mL). The thyroid function test indicated thyroid-stimulating hormone (TSH) levels at 0.1525 μIU/mL. B-ultrasonography of the thyroid showed heterogeneous cystic nodules. There was no evidence of pulmonary or any other local fungal infection. The brain MRI (Figures 2A,B) presented with noticeable meningeal thickening, large edema in the corpus callosum and white matter in both the hemispheres, and obvious ventricular expansion. In addition, MR hydrography of the cerebrospinal fluid indicated that the interventricular foramen of both the lateral ventricles was occluded, middle cerebral aqueduct was significantly narrowed, and the left lateral ventricle exhibited the most hydrocephalus in comparison with the other dilated ventricles. The lung CT (Figure 2C) showed no significant change as compared to the previous imaging. We began osmotherapy therapy after his admission. Mannitol and glycerol fructose were administered intravenously alternately every 6 h. However, the patient unexpectedly had a seizure 4 days after the admission. We treated him with diazepam and valproate in addition to the anti-fungal drug, liposomal amphotericin B. He was also treated with 20 % mannitol every 4 h. Nevertheless, he was imminently transferred to the neurosurgery department and a left lateral ventricle puncture and drainage surgery was performed. His CSF analysis demonstrated a normal white blood cell count (3/μL, normal < 8/μL) and sugar, protein, and chloride levels were also within the normal range. The percentages of polymorphonuclear cells, lymphocytes, and monocytes were not analyzed. The immunoglobulin analysis in the CSF revealed increased IgG levels (69.3 mg/L, normal range 4.80–58.60 mg/L), and decreased microalbumin levels (112 mg/L, normal range 139.0–246.0 mg/L). Additionally, CSF culture was performed, but no fungus and anaerobic bacteria were observed, and no acid-resistant bacilli were found in the smear. A few hyphae and spores were detected in the CSF under a fluorescence microscope with calcofluor white (CW) staining. A brain tissue specimen was collected during surgery for histopathological assessment, which showed mycelia and fungal granuloma (Figures 3C–E). We also sequenced *CARD9* and found that the patient carried a novel homozygous mutation, which underlines life-threatening, invasive fungal infections in otherwise healthy individuals.

On the first day after the surgery, the head drainage tube and the catheter were both unobstructed, but condition of the patient deteriorated. The patient appeared to be in a coma with an accelerated heart rate of 120 beats per minute. The bilateral pupils were circular and equal in size, diameter of 2 mm, with no reflex. Moreover, his limbs were stiff and the Babinski's sign was positive, which indicated herniation of the brain. The blood gas analysis revealed respiratory alkalosis. Two days later, his CSF analysis showed an accelerated white blood cell count (17/μL, normal < 8/μL) and protein levels (1.62 g/L, normal 0.15–0.45 g/L), accompanied with normal sugar levels and chloride ions. The percentages of polymorphonuclear cells, lymphocytes, and monocytes were not analyzed. He was treated with liposomal amphotericin B and voriconazole. In the next few days, his blood routine examination indicated an elevated white blood cell count with mainly neutrophils. Furthermore, *Pseudomonas aeruginosa, Enterobacter cloacae*, and *Streptococcus* F group were cultured from his sputum. Serious pulmonary infection, pleural effusion and partial atelectasis (Figures 2D,E) resulted in repeated resuscitation from respiratory failure. However, deteriorating intracranial hypertension, ventricle enlargement, compression of brain stem, extensive intracranial infarction, and edema (Figures 2F,G) predicted a poor prognosis. Consequently, the patient's family decided to withdraw the therapy and transferred him to a local hospital. However, he eventually died, even with an active treatment at the subsequent hospitalization.

## Discussion

*Exophiala dermatitidis*, formerly known as *W. dermatitidis*, is a dark fungus from the phylum Ascomycetes (Order Chaetothyriales) that secretes melanin. It has been shown that *E. dermatitis* prefers to grow in environments rich in aromatic or toxic hydrocarbons (e.g., benzoyl benzene and xylene), high temperature, and water resources (5). Humid tropical and subtropical climates and high temperatures have been reported to be responsible for the increasing prevalence of the infection (6). The main virulent source of *E. dermatitidis* is the melanin located in its cell wall. Melanin is not only a typical feature, but also an indispensable factor in resisting the environmental stress. In fungal meningoencephalitis and granulomatous disease, the fungi are located in the giant cell surrounded by fibrosis and reactive gliosis. Melanin production interferes with microglia recognition and clearance of the brain parenchymal fungi, thus, leading to high mortality (7). In addition to melanin, other virulence factors include chitin synthase, formation of meristems, high temperature resistance, adhesion and hydrophobicity of the cell wall, extracellular products, acidic or basic secondary metabolites, and so on (8).

The fungus causes a number of clinical manifestations of phaeohyphomycosis, including skin and subcutaneous infections, sinus infections, eye infections, disseminated infections, or brain infections. In previous case reports, *E. dermatitis* has been demonstrated to colonize the respiratory tract in the patients with cystic fibrosis (9, 10), However, recent studies have reported infections not only in the immunodeficient patients, but in the immunocompetent individuals as well (11).

Cases of CNS infection caused by *E. dermatitis* have mostly originated in the Asian countries, especially in east Asia such as Japan and China, where a majority of the cases have been reported in healthy individuals (12). It can not only infect the skin and subcutaneous tissues, but also invade the CNS, demonstrating neurotropic characteristics and a high risk of mortality (12). It was previously thought that *E. dermatitis* infection has a local race-specificity as most of the fatal brain infection cases were reported in young Asians without a history of immune disorders. However, cases have now been reported in other countries as well, including the United States, India, and Brazil, indicating the global susceptibility to the infection (1). A few cases of invasive fungal infections have been associated with hereditary innate immune disorders, such as *CARD9* and *STAT1* deficiency (13–15). The report by Lanternier (13) related *CARD9* deficiency specifically to *E. dermatitis*. Loss of *CARD9* gene function has been established to alter the bactericidal capacity of monocyte macrophages and microglia (16). The reported cases of CNS infection related to *CARD9* deficiency are summarized in Table 1.

**Table 1:** Reported patients with CNS infection related to *CARD9* mutation.

**Patient**	**Origin**	**Sex (status)**	**Familial/sporadic**	**Mutation**	**Fugus**	**Neurological abnormalities in clinical manifestations**	**References**
P1	Algeria	M (died)	Familial (Q289X)	NA (a patient in his family with fungal skin inflection carring a Q289X mutation of *CARD9*)	*Trichopython. violaceum*	Seizure and cerebral abscesses on CT scan	(17)
P2	Angola	F (alive)	Familial (R18W)	R18W/R18W	*Exophiala. dermatitidis*	No clinical neurological signs, 13 cerebral lesions on brain MRI	(17)
P3	Asian	F (alive)	NA	G72S/R373P	*Candida dubliniensis*	Meningoencephalitis	(17)
P4	El Salvador	F (alive)	Familial (R57H)	R57H/R57H	*Candida albicans*	Meningoencephalitis, osteomyelitis, obstructive hydrocephalus, brain abscesses	(17)
P5	French-Canadian	M (alive)	Familial (Y91H)	Y91H/Y91H	*Candida albicans*	Meningoencephalitis	(17)
P6	French-Canadian	M (alive)	Familial (Y91H,c.-529T>C)	Y91H/c.-529T>C	*Candida albicans*	Multiple intracranial cystic masses	(17)
P7	French-Canadian	F (alive)	Familial (Y91H,c.-529T>C)	Y91H/c.-529T>C	*Candida albicans*	Brain abscesses and vertebral osteomyelitis	(17)
P8		F (alive)	Familial (Y91H,c.-529T>C)	Y91H/c.-529T>C	NA	Lesions in the basal ganglia bilaterally, encephalomalacia	(17)
P9	Iran	M (died)	Familial (Q295X)	Q295X/Q295X	*Candida* spp.	Seizure, hydrocephalus, candida meningitis	(17)
P10		F (alive)		NA	*Candida* spp.	Brain tumor with severe skull destruction	(17)
P11		F (alive)		NA	*Candida* spp.	Candida meningoencephalitis	(17)
P12	Morocco	F (alive)	NA	Q289X/Q289X	*Candida albicans*	Papillary edema, a large perilesional edema with a mass effect on the left ventricle	(17)
P13	Iran	M (alive)	Familial (R35Q)	R35Q/R35Q	*Candida glabrata*	Brain abscess, meningoencephalitis	(17)
P14	Turkey	F (alive)	NA	R70W/R70W	*Candida albicans*	Brain abscess, meningitis	(17)
P15	Turkey	F (alive)	Familial (R70W)	R70W/R70W	*Candida albicans*	Brain leisions in MRI	(17)
P16	Turkey	M (alive)	Familial (R70W)	NA	*Candida albicans*	Cerebral Candidiasis, encephalitis	(17)
P17	Turkey	M (alive)	Familial (Q295X)	Q295X/Q295X	*Candida albicans*	Meningoencephalitis	(17)
P18		M (alive)		Q295X/Q295X	*Candida albicans*	Encephalitis	(17)
P19	Turkey	F (alive)	Familial (Q295X)	Q295X/Q295X	*Candida albicans*	Encephalitis	(17)
P20	Turkey	F (alive)	Familial (Q295X)	Q295X/Q295X	*Candida albicans, Aspergillus (indistinguishable)*	Bilateral brain lesions in MRI and	(17)
P21	Mix European	M (alive)	Familial (Q295X)	Q295X/Q295X	*Candida albicans, A.fumigatus*	Cerebral aspergillosis	(17)
P22	Algeria	F (alive)	Familial (Q289X)	Q289X/Q289X	*Trichophyton rubrum*	Right cerebral abscess	(18)

*P, patient; NA, not available; F, female; M, male; MRI, Magnetic Resonance Imaging*.

Considering that the parents of our patient are close relatives, we performed *CARD9* gene testing in his family, including his parents and younger sister (Figure 3A). Our patient carried a homozygous c.759 duplication (dup) mutation (chr9:139265021) in exon 5 of *CARD9* (RefSeq accession number NM_052813), resulting in frame shift of lysine to glutamate at position 254 and prematurely terminated transcription in the following 81 amino acids. The patient's healthy parents and his sister were found to be heterozygous for the mutation (Figure 3B). The segregation of the mutation was consistent with autosomal recessive (AR) *CARD9* deficiency with complete clinical penetrance. The genetic segregation pattern of *CARD9* in the patient was autosomal recessive, and the mutation carried by the patient was a homozygous locus. At present, we did not find any clinical significance of this mutation in the literature and public databases, so we believe that the mutation in *CARD9* in the patient is a pathogenic mutation. Further studies should be performed to investigate the functional changes from this novel mutation in *CARD9* and the potential molecular mechanisms in the disease pathogenesis.

Cerebral phaeohyphomycosis manifests as a single brain abscess (87%), meningitis (9%), encephalitis (2%), myelitis (2%), or arachnoiditis (1%) (19). The clinical symptoms are diverse and similar to any brain tissue damage, such as epilepsy, headache, brain irritation symptoms, fever, mental symptoms, etc. Hemiplegia and partial sensory disturbances, similar to stroke and tumor, are the most common symptoms, and are accompanied by symptoms more typical of infections such as headache and fever. Headache accounts for 55 % of the common clinical findings (20).

With no classic or pathognomonic imaging findings, different imaging investigations can make a difference between diagnosis and differential diagnosis. Intracranial proliferative lesions can be manifested as focal ring-enhanced lesions on CT and ring-shaped enhancement effect on magnetic resonance imaging (MRI) as the disease progresses. Intracranial infection manifestations such as meningeal thickening and intracranial hypertension suggested by ventricular enlargement and edema around the ventricles are also some of the common imaging findings (1, 2). The MRI presentation of our patient was mainly characterized by marked ventricular enlargement, ablocked cerebral aqueduct and extensive meningeal thickening.

Cerebrospinal fluid discoloration has been reported to be a unique feature if the meninges are involved in the pathology. Moreover, the CSF often appears black in *Exophiala* spp. infection. If the infection has not invaded the cerebrospinal membrane, the CSF test results are usually abnormal with hypoglycemia, mildly elevated protein concentration, sometimes accompanied by eosinophilia, and peripheral blood eosinophilia (21). The final diagnosis relies on appropriate pathology and culture assessments. In addition, polymerase chain reaction (PCR) can be carried out on tissue or fungal organisms to classify the fungus based on characteristic DNA sequences (2). The first CSF analysis in this patient was normal, and the CSF culture revealed no fungi growth, though fungal microscopy was positive. We hold the opinion that the results may be correlated with the progression of the infection.

Considering the history of fungal lymphadenitis caused by *E. dermatitis*, a special neurotropic feature of *E. dermatitis*, fungal hyphae, and granuloma detected in the intracranial tissue by biopsy, a novel homozygous mutation of *CARD9*, we diagnosed the patient with an intracranial infection caused by the rare fungus *E. dermatitis*.

With respect to the treatment of *E. dermatitis*, oral itraconazole is the first-line drug for skin infections, subcutaneous nodules or mycobacterial diseases (22). Amphotericin B (AMB) has a good fungicidal effect on *E. dermatitis in vitro* and can be used to treat systemic phaeohyphomycosis caused by *E. dermatitis*. Further, studies have shown that liposomal amphotericin B (L-AMB) is superior to deoxycholic amphotericin B (D-AMB) in mouse models of *E. dermatitis* infections (22, 23). Fluconazole was an early drug utilized in systemic antifungal therapy, but has not been useful for E. dermatitits infections due to its relatively weak anti-fungal efficacy against the fungus. 5-Fluorocytosine synergizes with amphotericin B, itraconazole or posaconazole, which enhance the intracellular penetration and activity of 5-fluorocytosine (1).

Although *E. dermatitis* is susceptible to fluconazole, itraconazole, and posaconazole *in vitro*, its frequent recurrence hinders effective treatment. Studies have shown that amphotericin B and itraconazole have lower minimum inhibitory concentrations, but most patients show poor outcomes with these drugs due to less permeability in the CNS. In contrast, voriconazole and posaconazole have higher brain permeability; thus, these can intensify the treatment of a disseminated *E. dermatitis* infection. Posaconazole is not considered first line therapy, but may provide an alternative salvage therapy after failure of other anti-fungals as it seems to be a good salvage treatment after failure of other antifungal drugs (22). The combination of amphotericin B, 5-fluorocytosine, and itraconazole in the treatment of cerebral phaeohyphomycosis has shown improved survival rate (24).

In addition to antifungal therapy, surgery may provide a necessary adjunctive therapy. Local lesions and granulomas should be thoroughly debrided (2). The brain abscess must be drained, because thick wall of the abscess hinders drug penetration. However, evading the spread of the infection after the surgery requires special attention. Patients with intracranial hypertension may require timely surgical decompression and drainage to prevent cerebral herniation and ameliorate symptoms.

Our patient showed no local brain abscess lesions, and the imaging manifestation included ventricular expansion, extensively thickened meninges, cerebral edema, and herniation of the brain. Intensive osmotherapy, liposomal amphotericin B combined with voriconazole intravenous injections, and lateral ventricle drainage constitute the predominant therapeutic regimen. However, the symptoms of intracranial hypertension were severe in the patient at the time of admission, and the disease progressed rapidly with a poor therapeutic outcome.

## Conclusion

Here, we report the first case of a *CARD9*-deficient Chinese male who suffered from cerebral phaeohyphomycosis caused by *E. dermatitidis*. Such underlying inherited errors of immunity should be considered as a possible diagnosis in the cases of fungal infection of the CNS. Prompt and proper treatment is imperative to prevent further neurological damage, and a combination therapy with full course is recommended in this fungal infection. Notwithstanding, this rare infection still has a poor prognosis and high mortality rate and, thus, needs further investigations to better understand the molecular mechanisms of its pathogenesis.

## Data Availability

The datasets generated for this study are available on request to the corresponding author.

## Ethics Statement

All the participants or their legal guardians provided written informed consents. This study was carried out in accordance with the recommendations of Ethics Committees of Union Hospital affiliated to Tongji Medical Collage of Huazhong University of Science and Technology. The written informed consents for the publication of this case report, including identifiable data from the subject and legal guardians were obtained in accordance with the Declaration of Helsinki.

## Author Contributions

CW and HX wrote the manuscript and prepared the figures. XJ performed the surgery and provided the clinical care. JZ cultured and identified the causative fungi. ZL explained the *CARD9* gene mutation and conducted genetic and genealogical studies. JC and YW critically reviewed and edited the manuscript. All the authors were involved in the work-up of the patient, planning, and conducting investigations.

### Conflict of Interest Statement

The authors declare that the research was conducted in the absence of any commercial or financial relationships that could be construed as a potential conflict of interest.
